# Supplementary material for: New Genomic Regions Identified for Resistance to Spot Blotch and Terminal Heat Stress in an Interspecific Population of Triticum aestivum and T. spelta
Source: Plants (Basel). 2022 Nov 5;11(21):2987. doi: 10.3390/plants11212987 (PMC9657703; doi:10.3390/plants11212987)
Supplement: Supplementary file 1 [file plants-11-02987-s001.zip › Supplementary Figures.pdf]

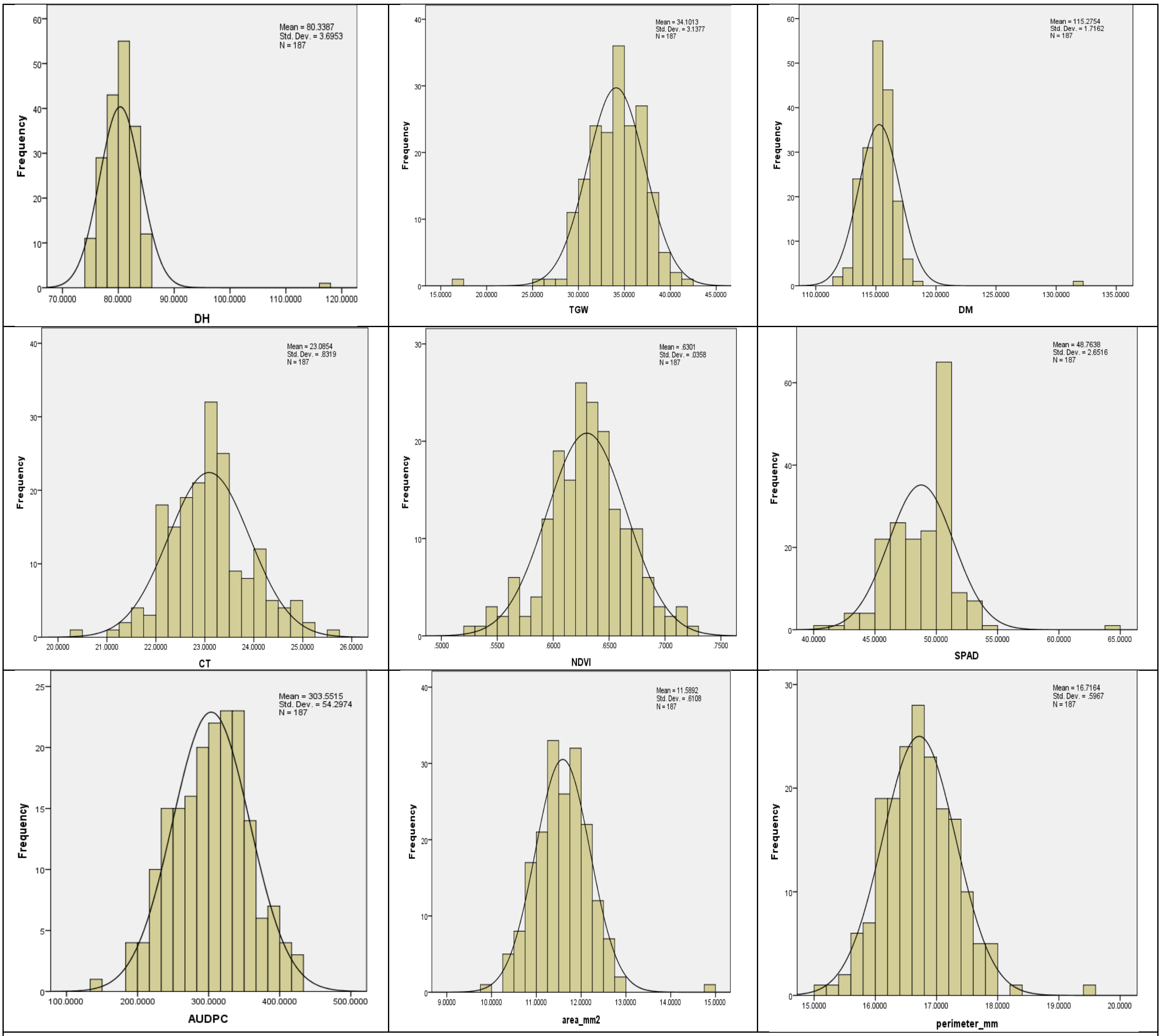

Figure S1 Frequency distribution of various phenotypic traits among RILs along with its parents under control (without inoculation) condition.

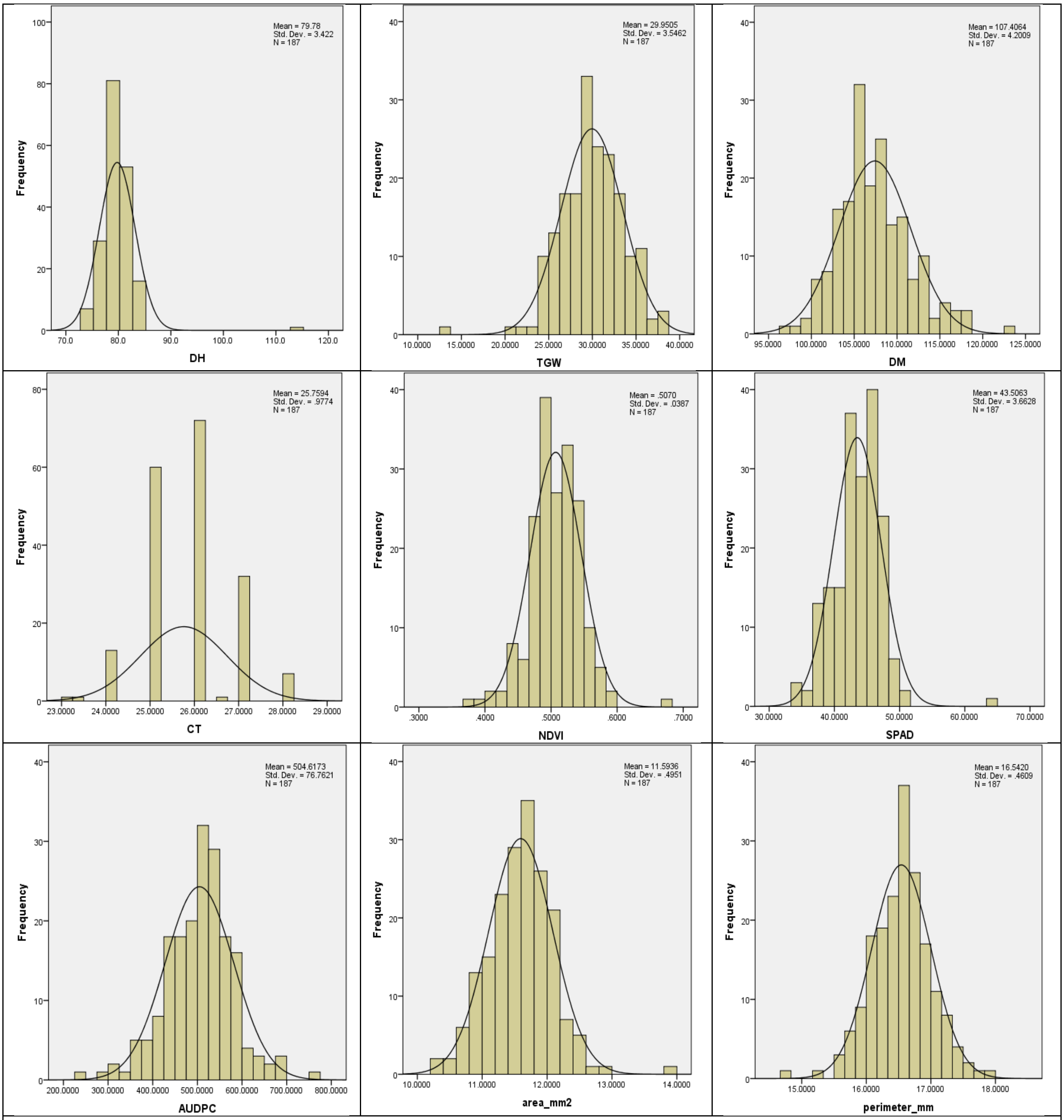

Figure S2 Frequency distribution of various phenotypic traits among RILs along with its parents in response to spot blotch

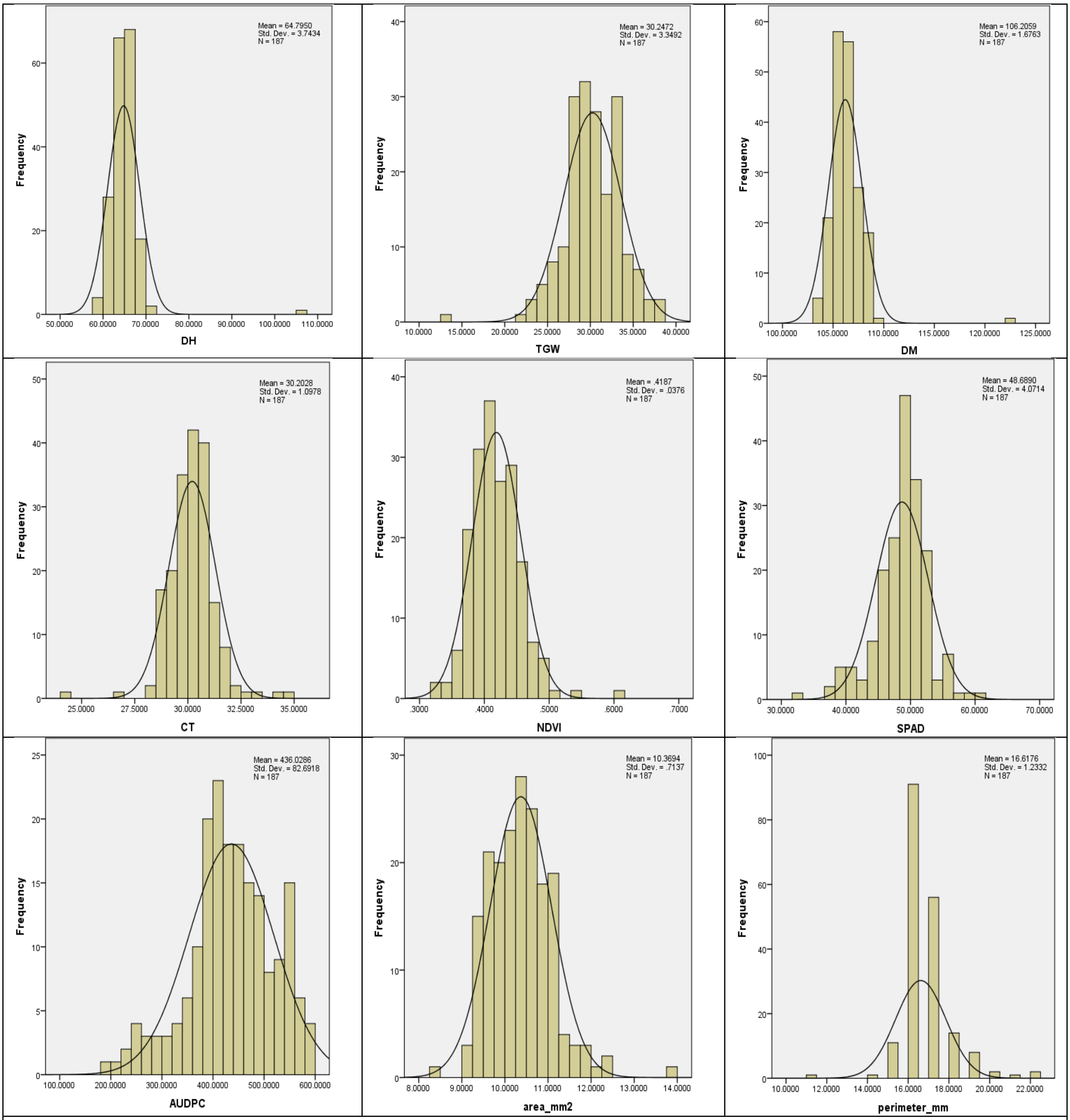

Figure S3 Frequency distribution of various phenotypic traits among RILs along with its parents under terminal heat stress

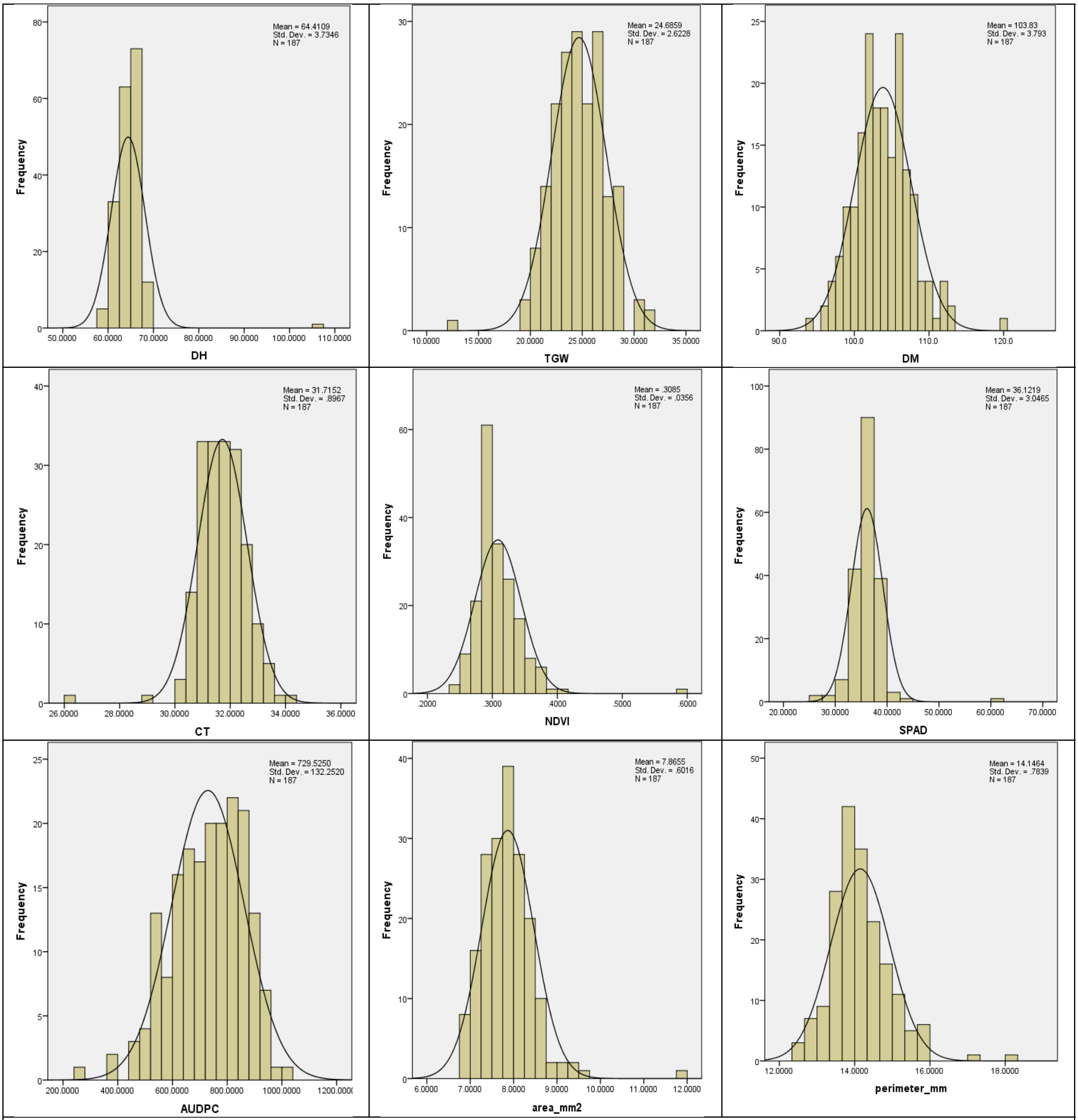

Figure S4 Frequency distribution of various phenotypic traits among RILs along with its parents under combined stress of spot blotch and terminal heat stress.

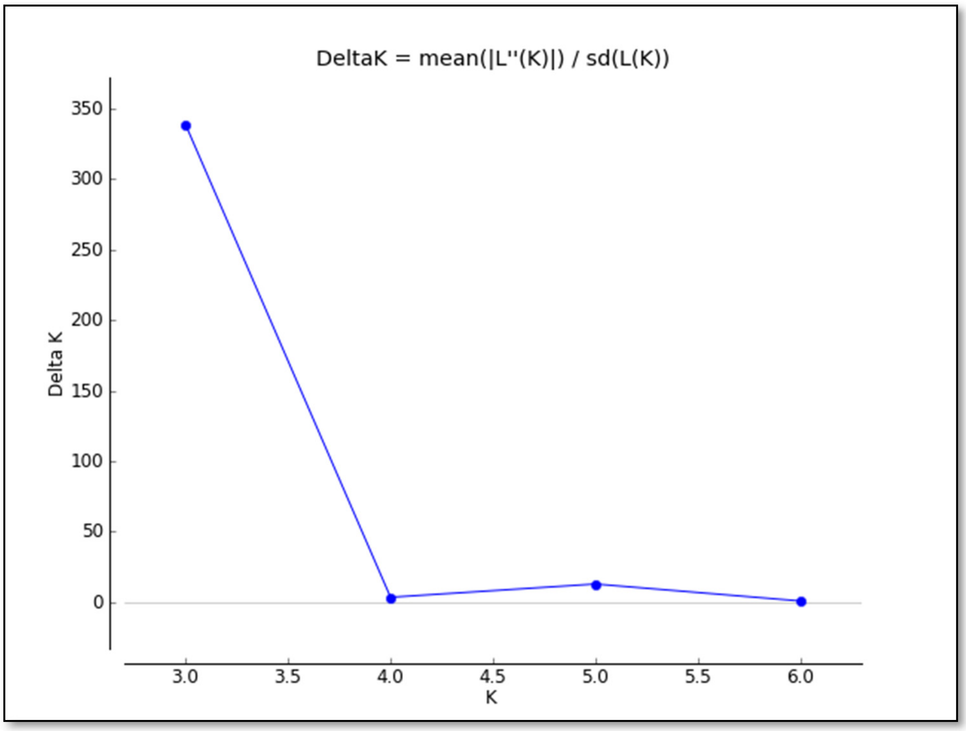

Figure S5 The plot of K versus Delta K showing variations (The steep change in slope indicates K=3 as the best choice for the number of clusters)

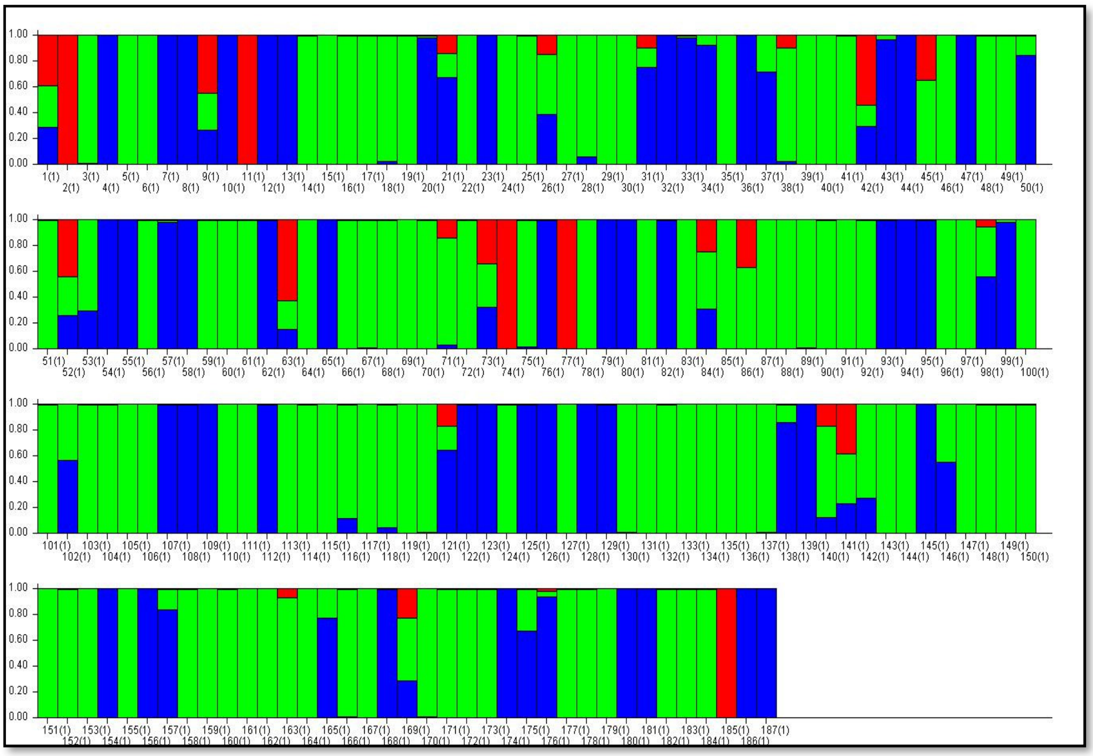

Figure S6 The plot showing the population structure of different recombinant inbred lines (RILs) along with parents in clusters for k=3. (The numbers on the horizontal axis are the line numbers)

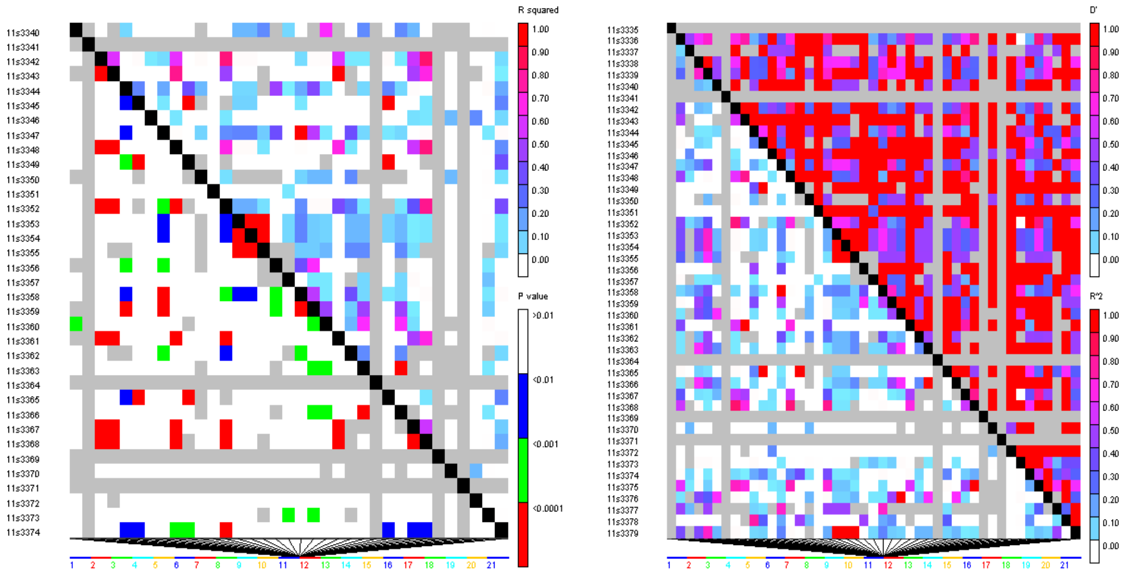

Figure S7 Linkage disequilibrium (LD) plot based on Kinship matrix and SNP markers

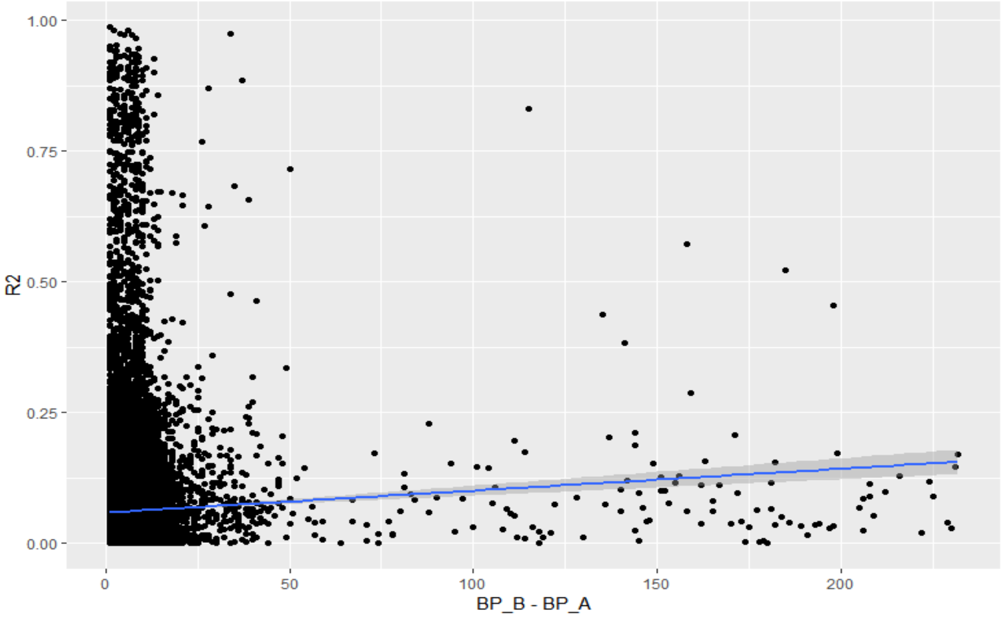

Figure S8 Linkage disequilibrium decay plots are displaying r^2 vs genetic distance (cM) in 185 RILs along with parents. LD was calculated from intra chromosomal pairs of the marker for whole genome with 95 percentile confidence.

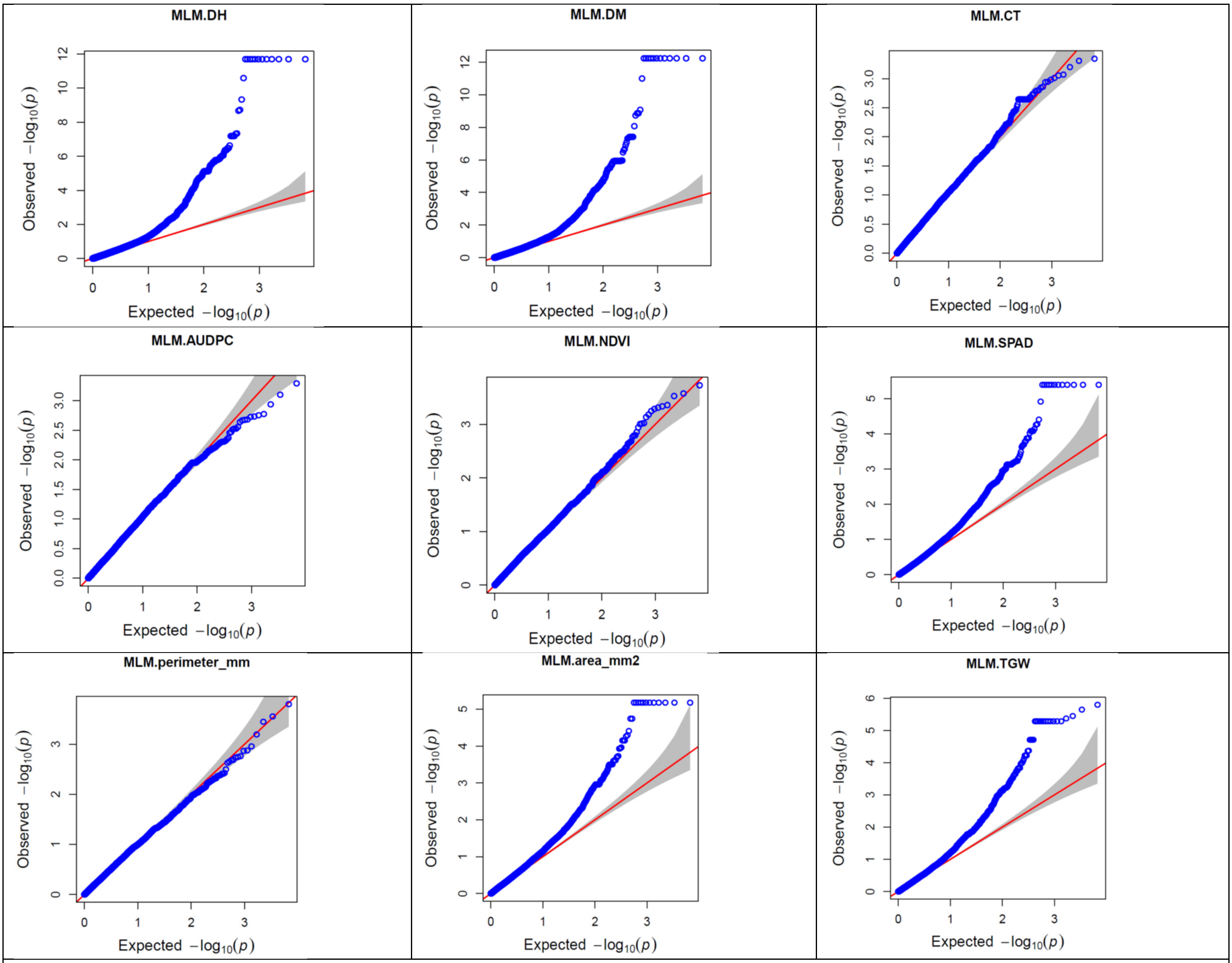

Figure S9 Quantile –Quantile (Q-Q) plot showing the distribution of the recombinant inbred lines (RILs) analyzed in multiple linear models.
