# Supplementary material for: New Genomic Regions Identified for Resistance to Spot Blotch and Terminal Heat Stress in an Interspecific Population of Triticum aestivum and T. spelta
Source: Plants (Basel). 2022 Nov 5;11(21):2987. doi: 10.3390/plants11212987 (PMC9657703; doi:10.3390/plants11212987)
Supplement: Supplementary file 1 [file plants-11-02987-s001.zip › Supplementary Tables.pdf]

Table S1: Mean performance for parent and recombinant inbred lines (RILs) across various environments and treatments

Trait			Environment and Treatments			
			Control (Timely sown protected)	Spot Blotch (Timely Sown Inoculated)	Terminal Heat stress (Late sown protected)	Spot blotch + Terminal Heat (Late sown Inoculated)
**DH (days)**	HUW234	Mean ± SD	74.5 ± 1	75.5 ± 1.5	59 ± 2.8	60.5 ± 2
	H+26	Mean ± SD	116 ± 1.8	114 ± 2	105 ± 2.3	105 ± 1.8
	RIL	Mean ± SD	80.18 ± 2.58	79.64 ± 2.26	64.61 ± 2.27	64.21 ± 2.24
		Range	74.17 - 85.83	74.5 - 85	58 - 72	58 - 69
**TGW (g)**	HUW234	Mean ± SD	39.59 ± 1.6	29.415 ± 3.4	36.02 ± 1.6	26.375 ± 2.9
	H+26	Mean ± SD	16.7 ± 2.5	12.7 ± 1.4	13.7 ± 3.1	12.07 ± 2.6
	RIL	Mean ± SD	34.17 ± 2.85	30.05 ± 3.33	30.31 ± 3.11	24.75 ± 2.46
		Range	25.32 - 41.32	21.08 - 38.19	21.65 - 38.58	19.41 - 31.55
**DM (days)**	HUW234	Mean ± SD	112.66 ± 2.6	107.16 ± 3.2	105.66 ± 2.65	99.5 ± 3.7
	H+26	Mean ± SD	132 ± 1.2	123 ± 3.7	122 ± 2.32	120 ± 4.2
	RIL	Mean ± SD	115.2 ± 1.19	107.32 ± 4.06	106.12 ± 1.22	103.76 ± 3.57
		Range	111.83 - 118.17	96.67 - 117.67	103.17 - 109.17	94.5 - 112.83
**CT (ºC)**	HUW234	Mean ± SD	22.18 ± 1.1	26.66 ± 0.89	30.52 ± 0.83	31.72 ± 0.91
	H+26	Mean ± SD	20.34 ± 0.79	23.34 ± 1.2	24.34 ± 0.9	26.04 ± 1.1
	RIL	Mean ± SD	23.11 ± 0.81	25.73 ± 0.95	30.23 ± 1.01	31.75 ± 0.8
		Range	21.09 - 25.58	23.37 - 28.3	26.78 - 34.85	28.99 - 34.12
**NDVI**	HUW234	Mean ± SD	0.60 ± 0.06	0.37 ± 0.09	0.41 ± 0.06	0.29 ± 0.1
	H+26	Mean ± SD	0.67 ± 0.037	0.67 ± 0.1	0.61 ± 0.82	0.59 ± 0.05
	RIL	Mean ± SD	0.63 ± 0.04	0.51 ± 0.04	0.42 ± 0.04	0.31 ± 0.03
		Range	0.52 - 0.72	0.39 - 0.59	0.33 - 0.54	0.24 - 0.41
**SPAD**	HUW234	Mean ± SD	49.88 ± 3.22	34.725 ± 4.23	46.84 ± 4.41	28.01 ± 3.82
	H+26	Mean ± SD	64.95 ± 4.11	64.95 ± 3.98	60.95 ± 3.94	60.05 ± 2.47
	RIL	Mean ± SD	48.66 ± 2.4	43.43 ± 3.25	48.63 ± 3.99	36.04 ± 2.43
		Range	40.35 - 53.53	34.18 - 50.46	31.74 - 58.77	26.1 - 43.34
**AUDPC**	HUW234	Mean ± SD	286.73 ± 68.62	765.30 ± 52.98	292.75 ± 102.39	904.87 ± 119.23
	H+26	Mean ± SD	142.84 ± 22.39	230.84 ± 44.23	189.84 ± 91.35	255.84 ± 102.92
	RIL	Mean ± SD	304.51 ± 53.27	504.69 ± 71.97	438.13 ± 80.42	731.14 ± 127.64

		Range	187.66 - 431.4	299.31 - 689.35	210.77 - 598.79	384.88 - 1022
**Grain area (mm^2)**	HUW234	Mean ± SD	12.17 ± 0.10	11.91 ± 0. 23	11.95 ± 0.54	7.91 ± 0.29
	H+26	Mean ± SD	14.87 ± 0.19	13.87 ± 0.36	13.87 ± 0.61	11.87 ± 0.41
	RIL	Mean ± SD	11.57 ± 0.56	11.58 ± 0.47	10.34 ± 0.66	7.84 ± 0.53
		Range	9.8 - 12.85	10.21 - 13	8.47 - 12.3	6.8 - 9.65
**Grain perimeter (mm)**	HUW234	Mean ± SD	17.16 ± 0.31	14.74 ± 0.13	17.04 ± 0.55	12.41 ± 0.26
	H+26	Mean ± SD	17.84 ± 0.81	17.84 ± 0.23	17.44 ± 0.91	14.84 ± 0.46
	RIL	Mean ± SD	16.71 ± 0.59	16.54 ± 0.43	16.62 ± 1.21	14.15 ± 0.78
		Range	15.06 - 19.5	15.2 - 17.78	15.06 - 19.50	12.45 - 18.04

Table S2: Correlation coefficients between different nine phenotypic traits using pooled different sowing dates and treatment

	DH	TGW	DM	CT	NDVI	SPAD	AUDPC	area_mm	perimeter_mm
DH	1	0.330*	0.546*	- 0.734*	0.725*	0.237*	- 0.395*	0.243*	0.127*
TGW	0.330*	1	0.389*	- 0.403*	0.452*	0.388*	- 0.504*	0.528*	0.452*
DM	0.546*	0.389*	1	- 0.509*	0.62*	0.304*	- 0.561*	0.268*	0.209*
CT	- 0.734*	- 0.403*	- 0.509*	1	- 0.510*	- 0.268*	0.362*	- 0.292*	- 0.194*
NDVI	0.725*	0.452*	0.62*	- 0.510*	1	0.467*	- 0.555*	0.181*	0.086*
SPAD	0.237*	0.388*	0.304*	- 0.268*	0.467*	1	- 0.451*	0.173*	0.085*
AUDPC	- 0.395*	- 0.504*	- 0.561*	0.362*	- 0.555*	- 0.451*	1	- 0.440*	- 0.400*
area_mm	0.243*	0.528*	0.268*	- 0.292*	0.181*	0.173*	- 0.440*	1	0.954*
perimeter_mm	0.127*	0.452*	0.209*	- 0.194*	0.086*	0.085*	- 0.400*	0.954*	1

* significant at p<0.0001 DH: Days to heading, TKW: Thousand Kernel weight DM: Days to Maturity, CT: Canopy Temperature, NDVI: Normalized Distributed Vegetative Index, AUDPC: Area Under Disease Progress Curve, SPAD: Soil Plant Analysis Development

Table S3: Summary of number, minor allele frequency (MAF) and density of single nucleotide polymorphism (SNP) markers used

	Chromosomes	No. of markers	Chromosome length (Mb)	Minor allele frequency	Average distance between SNPs (kb)
Genome A	1	339	594.10	0.2219	1753
	2	437	780.80	0.2369	1787
	3	384	750.84	0.1993	1955
	4	260	744.59	0.2381	2864
	5	358	709.77	0.2128	1983
	6	329	618.08	0.2517	1879
	7	517	736.71	0.2290	1425
Total (1-7)		**2624**			
Genome B	1	517	689.85	0.1919	1334
	2	493	801.26	0.2129	1625
	3	458	830.83	0.2529	1814
	4	140	673.62	0.1297	4812
	5	392	713.15	0.2114	1819
	6	393	720.99	0.2617	1835
	7	448	750.62	0.2341	1675
Total (1-7)		**2841**			
Genome D	1	145	495.45	0.234696	3417
	2	177	651.85	0.1722	3683
	3	151	615.55	0.1961	4077
	4	58	509.86	0.1655	8791
	5	115	566.08	0.1392	4922
	6	116	473.59	0.2060	4083
	7	142	638.69	0.2076	4498
Total (1-7)		**904**			

Table S4: The Evanno table output at different values of K

K	Reps	Mean LnP (K)	Stdev LnP (K)	Ln'(K)	|Ln''(K)|	Delta K
2	3	- 857197.66	43.97	—	—	—
3	3	- 788450.66	147.03	68747.00	49710.13	338.09
4	3	- 769413.80	2254.68	19036.86	8176.73	3.62
5	3	- 758553.66	3202.42	10860.13	41646.70	13.00
6	3	- 789340.23	80238.60	- 30786.56	80990.03	1.00
7	3	- 739136.76	11369.24	50203.46	—	—

Table S5: Linkage disequilibrium (LD) for the whole, A, B, and D genomes of wheat

Dataset	No. of markers	Total marker pairs	Mean of r2 for all pairs	Total unlinked pairs	Significant Pairs		
					Total (P<0.001)	Linked (r2 >0.1)	Un linked (r2 <0.1)
**Whole genome**	2639	129275	0.435	81786	32221	23996	81786
**A**	1120	54725	0.0627	38906	20520	9895	38906
**B**	1097	53575	0.1827	24854	23718	22368	24854
**D**	422	19825	0.1030	6910	4313	3792	6910

Table S6: SNPs associated with spot blotch resistance identified through GWAS in the 185 RILs from the cross of *Triticum aestivum* (HUW 234) and *T. spelta* (H+26)

Trait	SNP	Chromosome	Position	p-value	Minor Allele frequency (MAF)	r2	marker effect
Days to heading (Days)	1125940|F|0	1A	3495	8.20E-16	0.00535	0.4503	-0.1443
	1395486|F|0	1B	3286	8.20E-16	0.00535	0.4503	-0.5206
	2256281|F|0	3A	3303	8.20E-16	0.00535	0.4503	0.01125
	980238|F|0	3A	3508	8.20E-16	0.00535	0.4503	-0.5919
	1050819|F|0	4D	3482	8.20E-16	0.00535	0.4503	0.75167
	1029559|F|0	5B	3274	8.20E-16	0.00535	0.4503	0.61398
	1020582|F|0	5B	3486	8.20E-16	0.00535	0.4503	0.32027
	1126383|F|0	5B	6315	8.20E-16	0.00535	0.4503	-0.3855
	987983|F|0	5D	3530	8.20E-16	0.00535	0.4503	-0.3756
	2266275|F|0	6B	3267	8.20E-16	0.00535	0.4503	-0.2655
	2278379|F|0	7B	4429	8.20E-16	0.00535	0.4503	-0.1621
	1077356|F|0	2A	3516	1.60E-10	0.0107	0.28753	-0.3125
	983670|F|0	3A	3509	1.60E-10	0.0107	0.28753	-0.0778
	976829|F|0	3B	3484	1.60E-10	0.0107	0.28753	0.4934
	987210|F|0	6B	3574	1.60E-10	0.0107	0.28753	0.15896
	1021511|F|0	7A	4329	1.60E-10	0.0107	0.28753	-0.2651
	2275693|F|0	3A	3308	2.80E-10	0.5	0.28079	-0.3652
Days to maturity (Days)	1125940|F|0	1A	3495	1.00E-16	0.00535	0.48812	-0.0619
	1395486|F|0	1B	3286	1.00E-16	0.00535	0.48812	-0.2886
	2256281|F|0	3A	3303	1.00E-16	0.00535	0.48812	0.13197
	980238|F|0	3A	3508	1.00E-16	0.00535	0.48812	-0.4385
	1050819|F|0	4D	3482	1.00E-16	0.00535	0.48812	0.30905
	1029559|F|0	5B	3274	1.00E-16	0.00535	0.48812	0.3297
	1020582|F|0	5B	3486	1.00E-16	0.00535	0.48812	0.15955
	1126383|F|0	5B	6315	1.00E-16	0.00535	0.48812	-0.39
	987983|F|0	5D	3530	1.00E-16	0.00535	0.48812	-0.1065

	2266275|F|0	6B	3267	1.00E-16	0.00535	0.48812	-0.0225
	2278379|F|0	7B	4429	1.00E-16	0.00535	0.48812	-0.2085
	1077356|F|0	2A	3516	3.90E-11	0.0107	0.31577	-0.1509
	983670|F|0	3A	3509	3.90E-11	0.0107	0.31577	-0.0931
	976829|F|0	3B	3484	3.90E-11	0.0107	0.31577	0.08726
	987210|F|0	6B	3574	3.90E-11	0.0107	0.31577	0.02065
	1021511|F|0	7A	4329	3.90E-11	0.0107	0.31577	-0.1036
	2275693|F|0	3A	3308	2.50E-10	0.5	0.29282	-0.0244
AUDPC	3064429|F|0	5B	2883	0.00044	0.49733	0.05298	-0.6564
	1088945|F|0	3D	3278	0.00096	0.5	0.04461	0.27412
	2281188|F|0	2A	5634	0.00132	0.4492	0.04121	3.1096
	3028841|F|0	2D	3906	0.00141	0.5	0.04047	-1.1446
	1039495|F|0	2A	2161	0.00154	0.4492	0.03954	-8.8813
Canopy Temperature (°C)	1034888|F|0	4A	3281	0.00037	0.5	0.07074	-0.0197
	1125940|F|0	1A	3495	0.00113	0.00535	0.05884	-0.0072
	1395486|F|0	1B	3286	0.00113	0.00535	0.05884	-0.1396
	2256281|F|0	3A	3303	0.00113	0.00535	0.05884	-0.0537
	980238|F|0	3A	3508	0.00113	0.00535	0.05884	0.05024
NDVI	1029767|F|0	5A	6013	0.00028	0.32888	0.07355	0.00156
	2253029|F|0	2A	3116	0.00047	0.5	0.06813	-0.0045
	995480|F|0	6B	3454	0.00049	0.13102	0.0676	-0.0039
	1079395|F|0	7B	2832	0.00058	0.01604	0.0658	0.00476
	3064380|F|0	5A	4170	0.0007	0.12299	0.06392	-0.0024
	3064765|F|0	1B	928	0.00077	0.34225	0.06292	-0.0024
	1045022|F|0	5A	2928	0.00088	0.02139	0.06143	-0.0033
	1058939|F|0	1A	6063	0.00096	0.36364	0.0605	-0.0005
	1102573|F|0	1A	3376	0.00128	0.01337	0.05751	0.00978
SPAD	1125940|F|0	1A	3495	7.00E-07	0.00535	0.1764	-0.1124
	1395486|F|0	1B	3286	7.00E-07	0.00535	0.1764	-0.0305
	2256281|F|0	3A	3303	7.00E-07	0.00535	0.1764	-0.0626

	980238|F|0	3A	3508	7.00E-07	0.00535	0.1764	-0.089
	1050819|F|0	4D	3482	7.00E-07	0.00535	0.1764	0.45443
	1029559|F|0	5B	3274	7.00E-07	0.00535	0.1764	0.33204
	1020582|F|0	5B	3486	7.00E-07	0.00535	0.1764	0.78679
	1126383|F|0	5B	6315	7.00E-07	0.00535	0.1764	-0.3109
	987983|F|0	5D	3530	7.00E-07	0.00535	0.1764	-0.181
Test Weight (g)	2275693|F|0	3A	3308	1.10E-06	0.5	0.15883	-0.2261
	1125940|F|0	1A	3495	1.20E-06	0.00535	0.15753	0.18797
	1395486|F|0	1B	3286	1.20E-06	0.00535	0.15753	0.1447
	2256281|F|0	3A	3303	1.20E-06	0.00535	0.15753	-0.1669
	980238|F|0	3A	3508	1.20E-06	0.00535	0.15753	0.14922
	1050819|F|0	4D	3482	1.20E-06	0.00535	0.15753	-0.2597
	1029559|F|0	5B	3274	1.20E-06	0.00535	0.15753	-1.2436
	1020582|F|0	5B	3486	1.20E-06	0.00535	0.15753	-0.5015
	1126383|F|0	5B	6315	1.20E-06	0.00535	0.15753	0.30098
Grain Area (mm2)	1125940|F|0	1A	3495	9.10E-07	0.00535	0.14877	0.13395
	1395486|F|0	1B	3286	9.10E-07	0.00535	0.14877	0.01283
	2256281|F|0	3A	3303	9.10E-07	0.00535	0.14877	0.04926
	980238|F|0	3A	3508	9.10E-07	0.00535	0.14877	-0.0595
	1050819|F|0	4D	3482	9.10E-07	0.00535	0.14877	-0.0004
	1029559|F|0	5B	3274	9.10E-07	0.00535	0.14877	0.02607
	1020582|F|0	5B	3486	9.10E-07	0.00535	0.14877	0.05289
	1126383|F|0	5B	6315	9.10E-07	0.00535	0.14877	0.01694
	987983|F|0	5D	3530	9.10E-07	0.00535	0.14877	-0.042
Grain Perimeter (mm)	2280866|F|0	7A	6264	3.10E-05	0.00535	0.14654	0.05318
	3064641|F|0	3A	3335	4.90E-05	0.04545	0.14156	0.0421
	1241625|F|0	1B	5206	0.00017	0.13369	0.12841	0.28392
	1019339|F|0	2D	169	0.00028	0.33155	0.12341	-0.02
	1088359|F|0	5A	3195	0.0004	0.05882	0.1197	-0.0146
